# Patients’ research priorities and participation in primary ciliary dyskinesia research

**DOI:** 10.1136/bmjresp-2025-003364

**Published:** 2025-12-14

**Authors:** Yin Ting Lam, Laura Behan, Katie Dexter, Lucy Dixon, Claudia E Kuehni, Leonie Daria Schreck, Jane S Lucas, Myrofora Goutaki

**Affiliations:** 1 Institute of Social and Preventive Medicine, University of Bern, Bern, Switzerland; 2 Division of Respiratory Medicine, University Children's Hospital Zurich, Zurich, Switzerland; 3 School of Clinical and Experimental Medicine, Faculty of Medicine, University of Southampton, Southampton, UK; 4 PCD Support UK, London, UK; 5 Division of Paediatric Respiratory Medicine and Allergology, Department of Paediatrics, Inselspital, University Hospital, University of Bern, Bern, Switzerland

**Keywords:** Rare lung diseases, Surveys and Questionnaires, Systemic disease and lungs, Patient Outcome Assessment

## Abstract

**Introduction:**

People living with chronic diseases can provide a unique perspective for research that often differs from that of healthcare professionals. This is particularly important in rare diseases like primary ciliary dyskinesia (PCD), with many knowledge gaps and limited research resources. We aimed to assess participation of patients and caregivers in PCD research and identify their research priorities in a mixed-method study.

**Methods:**

We conducted in-depth, semistructured interviews with adults and adolescents with PCD, and caregivers of children with PCD. After verbatim transcription and thematic analysis, we developed an anonymous online survey, translated it into eight languages and circulated it widely in collaboration with PCD support groups worldwide and the European Lung Foundation.

**Results:**

The findings from the interviews identified key areas to be explored further through the survey including: developing treatments for PCD and increasing knowledge about different topics such as mental health, fertility, upper airway problems, treatment burden and impact of environment and lifestyle. 399 participants completed the online survey from 29 countries with median age 41 (IQR 33–49), 74% were female. 180 participants (45%) had participated in research before. For the remaining, the main reason for no participation was not being informed about studies (65%). 172 (43%) preferred regular research updates during a study. The top three ranked research priorities were (1) finding a cure to restore ciliary function; (2) developing treatments to improve lung function and reduce infections and mucus production; (3) finding the best way to manage the disease using existing medication. Other priorities were: involving more doctors and people with PCD in research, raising awareness of the condition and increasing knowledge about mental health and fertility.

**Conclusion:**

We found that people with PCD are motivated to participate in research when they are informed appropriately and invited. Their main research priorities relate to developing new treatments or improving the evidence base for existing treatments. Our findings will help the PCD research community to improve patient engagement in research and to draw common priorities together with the people who live with PCD and their families.

**WHAT IS ALREADY KNOWN ON THIS TOPIC**

- In rare diseases, such as primary ciliary dyskinesia (PCD), there are many knowledge gaps, but limited resources, and research priorities should take into consideration topics concerning patients.

**WHAT THIS STUDY ADDS**

- We found that people with PCD and their caregivers are motivated to participate in research when they are informed appropriately and invited, and they would like to receive research updates from studies in which they participate.
- Top-ranked research priorities relate to developing new treatments or improving the evidence base for existing treatments to help better manage the condition or to even cure their disease.

**HOW THIS STUDY MIGHT AFFECT RESEARCH, PRACTICE OR POLICY**

- By focussing on topics prioritised by people with PCD, participation in research may improve.
- Together with already published research priorities of healthcare professionals, our results will contribute to developing a common roadmap for future activities of the PCD research community.

## Introduction

 Primary ciliary dyskinesia (PCD) is a rare genetic, multi-organ disease with heterogenous clinical presentation.^1 2^ Foremost, PCD affects mucociliary clearance leading to chronic upper and lower respiratory symptoms and recurrent infections.^3^,^5^ In addition, it often affects other organ systems leading to heterotaxy or impaired infertility.^2 6^ Until now, most research was focused on pulmonary disease^7^,^12^ or on improving diagnosis of PCD, which remains challenging.^13^,^16^ Generally, PCD research activities were guided by what the research community found interesting and considered a priority, as in most diseases. Due to the heterogeneous nature of the disease, patients may develop questions in a wide range of topics. Their engagement in clinical research can ensure that research addresses important and relevant questions and prioritises patient-centred outcomes, leading to greater impact.^17^

In recent years, there have been many efforts in different diseases to explore research priorities jointly from the perspective of patients (mostly adults) and researchers across diseases.^18 19^ The process includes collecting and defining unanswered research questions and prioritising them by importance based on participants’ ranking. In other chronic respiratory diseases, large research networks assessed research priorities of patients and healthcare providers, underlining the value of the patient perspective.^20 21^

In the framework of the BEAT-PCD (Better Experimental Approaches to Treat PCD; beat-pcd.squarespace.com) clinical research collaboration, we explored research barriers and identified priorities in clinical and epidemiological research in the field of PCD from the perspectives of healthcare professionals and researchers.^22 23^ This study highlighted the need for a dedicated study focused on patients’ and caregivers’ perspectives. We hypothesised that people with PCD might prioritise different research questions compared with healthcare professionals, as was the case in other diseases.^24^ Therefore, we aimed to explore participation and identify priorities for PCD research from the perspective of patients aged ≥14 years and caregivers of children with PCD.

## Methods

### Study design

We performed a mixed-method study consisting of two phases: (1) semistructured in-depth interviews with people with PCD or caregivers of children with PCD and (2) an anonymous online survey based on results from phase 1.

### Phase 1: in-depth interviews

 We used purposive sampling to select interview participants among people registered in the Swiss PCD registry (CH-PCD).^25^ CH-PCD registers all people diagnosed with PCD living in Switzerland. We prioritised the invitations of CH-PCD participants who had previously and promptly participated in previous CH-PCD studies, independently of their participation in the local patient support group, assuming they had greater motivation and interest in research. We made sure to include participants from different age groups (adults, adolescents and caregivers of children) and with varying disease severity to collect rich and diverse data. Selected participants received an invitation and study information by post. Outside of Switzerland, all participants were engaged somehow with a patient support group, as we recruited volunteer participants who responded to our study call circulated by the PCD support groups in Spain and the UK and through the BEAT-PCD patient advisory group. Volunteers contacted us directly via email and received an invitation with detailed study information afterwards.

 In close collaboration with patient partners and representatives from PCD support groups, we developed an interview guide (supplemental document) in German and English for the in-depth, semistructured interviews. The interview guide was not prescriptive; instead, we encouraged participants to explore any area and followed new topics opportunistically to ensure depth of information. We conducted and recorded interviews using a teleconferencing software (Zoom) to facilitate notetaking and interview transcription, from 3 March 2021 to 17 January 2022. Before every interview, we obtained written and verbal informed consent. Participants had the option of having their cameras turned on or off. The first author (YTL) conducted all interviews, after she received training in-depth interviews. Her background as a paediatrician, with experience in talking to patients or parents of affected children, allowed her to notice reluctance or chance for conversational exploration and relevant behavioural cues. Further, her background impacted personal reflexivity and credibility with listening skills and taking time for the participants’ conscious pauses to contemplate, prompting going deeper into thoughts or exploring new topics. Being a physician and researcher in PCD might have put her in a power position regarding interpersonal reflexivity.^26 27^

 The purpose of phase 1 was to develop the online survey (phase 2), and the aim was not to achieve data saturation. Therefore, we aimed for a pragmatic sample size of 15–20 interviews, which would still allow us to collect rich and detailed data ensuring information power, especially for a rare disease like PCD.^28^ We transcribed all interviews verbatim focusing on protecting participants’ anonymity by removing identifying information. YTL and last author (MG), female senior researcher, expert in PCD and trained in qualitative research, coded the interviews inductively using a thematic analysis of Braun and Clarke approach using NVivo software - release 1.7.1.^29 30^ We enhanced trustworthiness of the data by review and confirmation of findings with other co-authors, LB who is a qualitative researcher, and KD and LD who were patient support group representatives.^27^ We followed a COREQ (Consolidated criteria for reporting qualitative research) checklist for reporting qualitative research throughout the study.^31^ Quotes were slightly edited for privacy and clarity and translated by YTL when needed. To ensure anonymity, we present quotes without details on sex or age, just by stating the participant group (adult, adolescent, parent) and a code used for pseudonymisation.

### Phase 2 – Online survey

Based on thematic analysis results of the interviews, we developed an anonymous online survey.^32^ Feedback was obtained from patient partners and experts in qualitative research and survey development. We set up the survey online in eight languages (English, French, German, Greek, Italian, Norwegian, Spanish, Turkish) in a Research Electronic Data Capture (REDCap) study database hosted by the clinical trials unit of the University of Bern.^33^ We chose the languages based on the participation numbers of people to a previous BEAT-PCD patient survey, choosing the most popular languages.^28^ We shared the survey link widely via PCD patient support groups worldwide and through the European Lung Foundation. Therefore, we also developed the survey in paper form in German and French and sent it by post to adults, adolescents and caregivers of children with PCD registered in CH-PCD. Participation was open from 26 June to 22 September 2023. For ranking questions, we used a 3-point, 5-point or 7-point Likert scale, ranging from ‘most important to you’ to ‘least important to you’.

We described characteristics of survey participants using median and IQR for continuous variables, numbers and proportions for categorical variables. We inquired about and described the median age of the person completing the survey who was either an adolescent or adult with PCD or a caregiver of a child with PCD. We studied possible predictors of staying informed about PCD research and participating in PCD research, including sex, living in a country with a support group, and participation in a support group, using separate multivariable logistic regression models. We ranked research priorities using the mean of a reciprocal ranking score (0–1) to assess the overall top three priorities.^34^ Each research question was scored with 1 if ranked first, 1/2 if ranked second, 1/3 if ranked third priority, and 0 if not ranked among the top three priorities. We hypothesised there might be different rankings by sex or relationship to PCD, and compared the rankings by sex, and separately by persons with PCD and parents/caregivers of children with PCD. A higher mean score indicated higher priority. We included participants with complete ranking questions. Participants with mainly missing answers were excluded. Missings were described as ‘not reported’. We reported based on the Strengthening the Reporting of Observational Studies in Epidemiology statement.^35^ We performed analyses using Stata version 15.1. We received approval from the Bernese cantonal ethics committee (KEK 2020-02250), Switzerland, for both phases of the study.

### Patient and public involvement

Patient partners were involved actively and as co-authors from the conception of the study throughout its whole duration, particularly due to the nature of our research questions. Patient partners as co-authors reviewed the interview guide and adapted it to more patient-friendly wording. They also contributed to selecting purposively possible interview candidates among their group members. At phase 2, patient partners reviewed content and wording of questions of the online survey and shared it via their communication channels. We worked and will continue working together with the BEAT-PCD patient advisory group and support groups worldwide to present results of this study in conferences and meetings. We will develop and distribute lay summaries of our findings.

## Results

### Participants

22 people agreed to be interviewed, surpassing our original target. They were 9 adults, 9 parents and 4 adolescents; 16 were female (73%). Median age was 25 years (IQR 27–51); 9 lived in Switzerland, 7 in the UK, 3 in other European countries and 3 in non-European countries. Interviews lasted from 45 to 120 min.

### Phase 1) in-depth interviews

**Figure 1:**
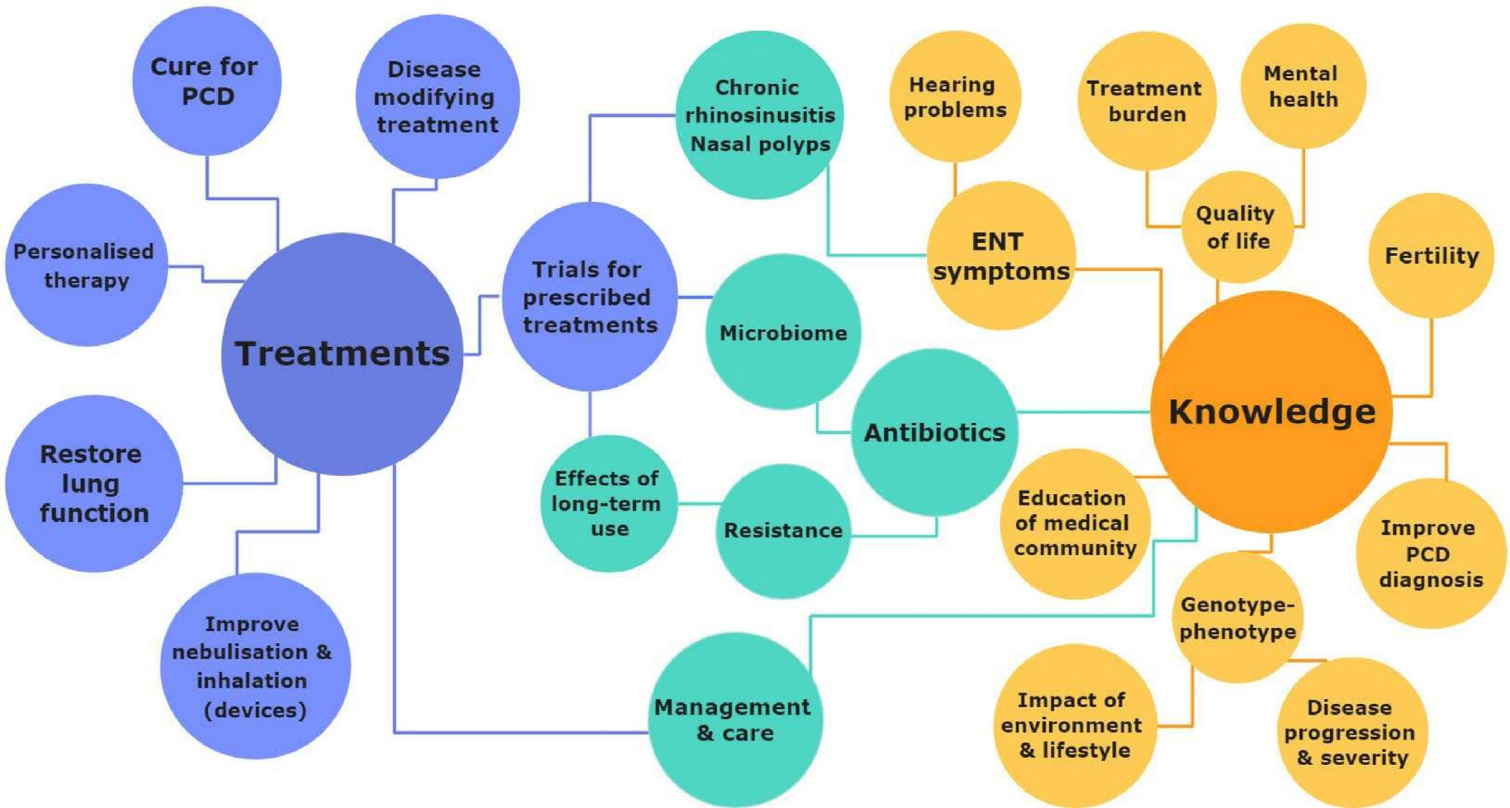
Main interview themes on research priorities for primary ciliary dyskinesia (PCD) suggested by people with PCD or caregivers of children with PCD. ENT, ear-nose-throat.

We identified two broad theme categories from the interviews: developing treatments and increasing knowledge in different fields (figure 1).

#### Developing treatments

Developing a cure for PCD or disease-modifying treatments, as well as personalised therapy or medications to restore lung function, were recurrent themes.

What I WANT is a PCD version for the new cystic fibrosis drug that apparently works! That’s what I WANT but that’s very difficult to create I’m going to say. … So if you could make a wonder drug that would be great! But obviously that’s very difficult to do. (Adolescent; Ea6)

Obviously, I’m REALLY interested in the possibility of genetic treatments for the condition. Ultimately, this seems like a condition which ought to be curable. […] But maybe one day … maybe by the end of my lifetime will be a point where we will be doing personalised genetic treatments for conditions. I wonder … it would be nice to think we will. (Adult; EA19)

Concerning treatments, further subthemes were restoring lung function and improving inhalation devices. Participants mentioned the need for clinical trials for prescribed treatments, questioning the appropriateness of their prescribed treatments, which are often modelled after treatments for cystic fibrosis (CF) without scientific evidence for PCD.

And have interventions you have like for … CF! We often model everything on CF, is that appropriate? (Adult; EA5)

Participants mentioned the effect of treatments on the microbiome and the need for development of new antibiotics. They worried about antibiotic resistance and long-term effects of prescribed treatments. Developing more effective treatments for nasal polyps was also highlighted.

#### Increasing knowledge

Increasing knowledge in several understudied areas of PCD was another broad theme. Participants requested increasing overall knowledge on ear-nose-throat (ENT) symptoms including hearing problems. When one participant heard about ongoing research on this topic, their reaction was:

But that’s fantastic to see that people are more looking into how ENT (symptoms) affect the patients. (Adult; EA5)

Other themes referred to the need to increase knowledge about the burden of disease as well as the treatment burden, and how both disease and treatment burden affect mental health and quality of life. Participants felt research into disease and treatment burden had been neglected.

Everybody with PCD, that I have spoken to, has had a HUGE impact on their ability to work. And yet this is nowhere (mentioned), right? This is not in the literature at all. We talk about disease burden, but you never look UP from the clinic … we never look up really from the patient. […] I feel … that my treatments are a burden, a hindrance. We don’t look at the lived ramifications of that, which is that many people are NOT working full time because of PCD. They’re STRUGGLING to work. Whether young or old … it seems to be a theme. I just don’t think it’s come up yet. (Adult; EA9)

Participants wished for studies to increase knowledge on fertility in PCD as well as improving PCD diagnosis. They also asked for better understanding of genotype-phenotype correlations, their associations with disease progression and the potential impacts of environmental factors and lifestyle choices.

Another important theme was increasing the awareness of PCD in the medical community, especially of general awareness for PCD, as patients or their family members often had to explain the disease to their doctors.

Because in the past, my mother took us to the emergency room, and she had to explain to the doctors what PCD was. Having to go through this process is NOT okay. I think the doctors MUST know what PCD is. I know it’s a rare disease and it’s not common. But this is not a reason for not knowing PCD. Doctors must know what PCD is, just as they know other diseases like cystic fibrosis. But nobody knows PCD. So we have to inform doctors about PCD ourselves. (Adolescent; Ea17)

An overarching theme related to both broad categories was improving PCD management and care by establishing specialised PCD care centres. We included a detailed list of themes and specific questions relating to each theme in the supplementary section under (online supplemental table 2).

### Phase 2) Online survey

399 participants completed the survey with median age 41 years (IQR 33–49), living in 29 different countries; 74% were female (table 1). Half (52%) had PCD themselves, of which 6 were adolescents aged 13–17 years. The others were caregivers of a child with PCD, diagnosed at a median age of 3 years (IQR: 1–7). 286 (72%) participants knew there was a PCD support group in their country of residence (online supplemental table S1). Among them, only half (51%) participated in activities or were representatives of the group. 86 (23%) participants living in countries with a national PCD support group did not know about its existence.

**Table 1:** Characteristics of survey participants (n=399)

	N (%)
Country of residence	
France	70 (17.5)
Germany	64 (16)
USA	42 (10.5)
Switzerland	40 (10)
Italy	38 (9.5)
UK	23 (6)
Spain	20 (5)
Australia	11 (3)
Other European countries	69 (17)
Other non-European countries	12 (3)
Not reported	10 (2.5)
Sex	
Female	295 (74)
Male	100 (25)
Not reported	4 (1)
Age of participants in median years (IQR)*	41 (33–49)
Connection to PCD	
Person with PCD	207 (52)
Caregiver of child with PCD	167 (42)
Not reported	25 (6)

Characteristics presented as N (%) or median (IQR) unless otherwise stated.

* Among 380 participants (person with PCD and caregivers of children with PCD) who reported their age. Age represents the age of the person completing the survey, not the person living with PCD.

PCD, primary ciliary dyskinesia.

Two thirds (67%) of participants said they remained informed about research related to PCD, mostly through word of mouth (42%) (table 2). About half (45%) had participated in PCD research before, of them 35% were invited by their physician to participate. The reasons for not having participated in PCD research before were most commonly not being informed about studies (65% of 168 participants) or not fitting the inclusion criteria (8%). Other reasons were that studies took place in different countries or more than 10 driving hours away, because participants were only recently diagnosed, or there had not been any opportunities yet.

**Table 2:** Experiences from previous participation in PCD research among survey participants (n=399)

	N (%)
Actively staying informed about PCD research	
Yes	268 (67)
No	25 (33)
Not reported	2 (1)
Methods to keep updated with PCD research*	
Word of mouth in person or online	113 (42)
National support group website or social media	36 (13)
BEAT-PCD or ERN-Lung websites	30 (11)
Support groups of other countries, website or social media	25 (9)
Online searches	22 (8)
Mailing list of support groups	12 (5)
Medical staff	8 (3)
European Lung Foundation or other respiratory patient support groups	4 (2)
Online library access	4 (2)
Online search alert	2 (1)
Other method	9 (3)
Not reported	3 (1)
Prior participation in PCD research	
Yes	180 (45)
No	168 (42)
I don’t know	24 (6)
Not reported	27 (7)
Method of prior invitation to participate in research†	
Invited by their treating physician	63 (35)
Invited by study group	32 (18)
Informed by support group	23 (13)
Followed study call on social media of support group	23 (13)
Enrolled in mailing list to receive research calls	15 (8)
Found study call on website of support group	15 (8)
Heard from other person with PCD	2 (1)
Other	6 (3)
Not reported	1 (1)
Reason for not having participated in PCD research before‡	
They were not informed about research studies	110 (65)
They did not fit in inclusion criteria	14 (8)
They did not receive enough study information	7 (4)
They were not interested in the research study	6 (4)
Study required a lot of time and effort	6 (4)
They only chose to participate if research benefited them directly	6 (4)
They worried about possible long-term effects of the research	5 (3)
Study required hospital visits	2 (1)
Other reasons	11 (6.5)
Not reported	1 (0.5)

Characteristics presented as N (%).

* Among 268 participants who keep updated with PCD research, multiple answers possible.

† Among 180 survey participants who had participated in PCD research before.

‡ Among 168 participants who have not participated in PCD research before.

BEAT-PCD, Better Experimental Approaches to Treat PCD – clinical research collaboration supported by the European Respiratory Society; ERN-Lung, European Reference Network; PCD, primary ciliary dyskinesia.

Participation in a national support group appears to have a positive effect on both in staying informed about PCD research (OR 1.4, 95% CI 0.9 to 2.3; online supplemental figure 1a), and on participating in PCD research (OR 1.5, 95% CI 1.0 to 2.4; online supplemental figure 1b). We did not find any association of staying informed about or participating in PCD research (online supplemental figure 1b) with being a person with PCD or a caregiver, man or woman, or living in a country with a PCD support group.

Among the 180 participants who had participated in PCD research before, about half (46%) had received study results (table 3). When received, results were understandable for most people. Participants preferred receiving regular updates by email or on a study website during the study duration (43%). Only 2% of participants were not interested in receiving any results. Participants preferred receiving feedback during the study (52%) compared with asking back about results (32%).

**Table 3:** Experiences and preferences about receiving feedback on PCD research results among survey participants (n=399)

	N (%)
Received study results after study completion*	
Yes, received results/updates without any efforts	70 (39)
Yes, but had to ask for results	13 (7)
No, but research is still ongoing	40 (22)
No, never heard back	36 (20)
Not sure	17 (10)
Not reported	4 (2)
If research results were received†	
They understood all information	53 (64)
They understood some parts	26 (31)
They did not understand most of the information	1 (1)
They do not remember	3 (4)
Preferred time to receive research results	
Receive regular updates and results during the study via email or on website	172 (43)
Receive final study results at the end of the study	88 (22)
Receive regular updates and results during the study online or on-site with research team	80 (20)
Not interested in receiving research results	8 (2)
Not reported	51 (13)
Preferred method to receive research results‡	
Study website	97 (24)
Email	75 (19)
Video	17 (4)
Letter	16 (4)
Lay summary	15 (4)
Scientific publication	5 (1)
Own result in addition	10 (3)
Not reported	164 (41)
Preferred to	
Receive feedback during research without actively requesting	207 (52)
Ask actively about research results when they wish	127 (32)
Not reported	65 (16)

Characteristics presented as N (%).

* Among 180 survey participants who had participated in PCD research before.

† Among 83 participants who had received research results.

‡ Multiple replies possible.

PCD, primary ciliary dyskinesia.

Among the research priorities participants were asked to rank, the top three priorities were: (1) “Can we find new medication that will ‘cure’ PCD or reduce the need for treatment by restoring the function of cilia in the body (like the medication available in cystic fibrosis)”; (2) “Are there treatments that will improve lung function, reduce infections, and reduce the amount of mucus I produce?”; and (3) “What is the best way to treat PCD (including lungs, ears, and nose) using existing medication and other management approaches?” (table 4). Ranking scores on overall top priorities varied, ranging from 0.04 to 0.48, highlighting that many topics were ranked highly by participants even if not included in the top of the list. Comparing research priorities by sex, men ranked research on life expectancy as more important and research on ENT symptoms as less important, compared with women. The top 10 research priorities were ranked similarly by persons with PCD and parents/caregivers of children with PCD; however, persons with PCD ranked research on mental health and fertility higher than parents (online supplemental table S3). In the final question, participants could suggest other research topics they considered a priority that were not already listed. Answers included: having genetic counselling for family planning and foetal genetic testing, defining which specific exercises can be done at home to manage one’s symptoms, and studying the effects of PCD on the sense of smell.

**Table 4:** Overall top research priorities for primary ciliary dyskinesia (PCD) as ranked by people with PCD or parents/caregivers of children with PCD, overall and by sex of survey participants

		Reciprocal mean score		
Rank	Research topics	Total (n=399)	Females (n=295)	Males (n=100)
1	Can we find new medication that will ‘cure’ PCD or reduce the need for treatment by restoring the function of cilia in the body (like the medication available in cystic fibrosis)	0.479	0.474	0.483
2	Are there treatments that will improve lung function, reduce infections, and reduce the amount of mucus I produce?	0.220	0.209	0.252
3	What is the best way to treat PCD (including lungs, ears, and nose) using existing medication and other management approaches?	0.204	0.192	0.213
4	Can we find new antibiotics or other medication to tackle antibiotic resistance, and what are the effects of long-term antibiotic use?	0.129	0.135	0.110
5	How can we get more doctors and people with PCD involved in research and make them aware of this condition?	0.127	0.125	0.130
6	What health-related behaviours can I do, and what everyday things should I avoid to control the improvement or worsening of my symptoms and quality of life?	0.094	0.077	0.133
7	How is mental health affected in people with PCD and their families (psychological aspects of PCD e.g. treatment burden and coping in daily life)?	0.074	0.082	0.053
8	What is the life expectancy in PCD and what are the long-term impacts of this disease and its treatments?	0.056	0.051	0.073
9	How is fertility affected in patients with PCD and what are the best fertility management approaches?	0.055	0.059	0.050
10	What is the best medication plan for each patient with PCD, including specific dosages and route (orally or intravenous)?	0.054	0.057	0.048
11	How should PCD be managed correctly in different age groups (also in people without symptoms)?	0.051	0.052	0.045
12	How does PCD affect the ears, balance issues, nose, and sinuses and how are these problems linked with problems in the lungs?	0.049	0.059	0.020
13	How does PCD affect the gastrointestinal system, such as stomach and intestines (e.g. reflux, indigestion, bloating)?	0.047	0.052	0.030
14	Are specific genes associated with specific symptoms or more severe disease (genotype-phenotype correlation)?	0.044	0.051	0.025
15	How can the treatment duration and treatment efforts be reduced without compromising effectiveness?	0.039	0.040	0.038

Research topics ranked from most to least important (listed among top three overall priorities) based on the mean of reciprocal ranking score (0–1); each question was scored with 1 if ranked first, 1/2 if ranked second, 1/3 if ranked third and 0 if not ranked among the top three priorities. 4 participants preferred not reporting their sex and were excluded from the stratified scores.

PCD, primary ciliary dyskinesia.

## Discussion

With a mixed-method design, we assessed participation of people with PCD and caregivers of children with PCD in research and identified research priorities from their perspectives. We found that people with PCD and caregivers of children with PCD were motivated to participate in PCD research, and almost half of them already had participated. Almost all would like to receive research updates from studies in which they participate. Top-ranked research priorities relate to developing new treatments or improving the evidence base for existing treatments to help better manage the condition or to even cure their disease. Our findings inform the PCD research community about opinions and preferences of patients regarding their participation in research and the communication of results. The study also highlights the importance of PCD support groups, which should be supported by healthcare professionals. Close collaboration of researchers with support groups will encourage further participation of people with PCD in upcoming research and facilitate wider dissemination of results in an appropriate format. The results of this study, taken together with the priorities identified by PCD professionals and researchers,^23^ will strengthen future research, especially of large research networks, such as BEAT-PCD, and contribute to ensuring that it does focus on questions most relevant to PCD patients and their families.

Ours is the first study to explore participation in research and research priorities for PCD from the perspective of patients as well as adolescent patients with PCD and caregivers of children with PCD. Performing semistructured interviews allowed us to explore patients’ perspectives in depth in order to develop a survey that included many different opinions. Our study was strengthened by the involvement of patient partners in all study steps, from the very beginning with the development of the interview guide to creating the survey. The majority of survey participants were female, which may have led to under-representation of male participants, a common phenomenon in online surveys.^36^ The survey was distributed during summer, which might have affected participation; however, the 3-month duration ensured any such effect was minimal. Our survey was circulated worldwide and was available in 8 languages ensuring wide reach (399 participants from 29 countries), but our study call reached only people with connections to PCD support groups, BEAT-PCD or the European Lung Foundation. People outside of this circle, without knowledge of the survey languages, or who had no ability to participate online could not participate.

We found collaboration between researchers and patient support groups to be an important factor that can contribute to research participation, which was also described in a systematic review of approaches for engaging patients for research on rare diseases.^17^ Closer collaboration with patient support groups can help disseminate study calls by using their available media of website, specific mailing list for study calls or newsletters, ensuring eligible patients are informed about research studies. Representatives from different patient support groups worldwide are actively involved in research initiatives, participate in patient advisory boards and help shape the direction of PCD research in a truly meaningful way. For example, several patient representatives are members of the management committee or the patient advisory group of the BEAT-PCD clinical research collaboration. Another example that showcases the importance of such partnerships is the Living with PCD study, which was set up together with representatives from different support groups and has a truly participatory design, where the patient advisory board contributes to developing future research topics.^17 18 21 37^

Preferably, participants would like to receive research updates during the study process and, most importantly, receive any feedback in an appropriate manner. We found at least one third of participants had not received any results from studies in which they had participated. If received, participants stated that results were mostly understandable, but understanding could further be improved with use of lay summaries co-developed with patient partners. Reporting results back in lay summaries is a growing effort among researchers to convey their results directly without misconstruction or misinterpretation.^38^ Preferred methods of receiving research results or feedback were digital media, for example, via newsletters, which facilitates active real-time public communication and research visibility benefitting future research participation.^39^ In a qualitative study with semistructured interviews with research participants and patient advisors about contributing to health research, the authors reported that communication between research teams, participants and clinicians could be improved, for example, by simplifying study documentation and providing feedback on findings.^40^ We also found this theme in our interviews along with improving knowledge and awareness of PCD in the medical community.

The highest ranked research priority was finding a cure for PCD; all top three ranked priorities were related to treatments to manage symptoms and improve quality of life. While these results are not surprising, they highlight the need for further clinical trials on the management of PCD as current evidence is limited.^41^,^43^ This study also confirms the enthusiasm and anticipation of people with PCD regarding the new potential molecular disease-modifying treatments that are in the pipeline for several disease-causing genes and the ongoing efforts of the medical community to provide access for patients to these ongoing trials.^44^,^46^ Interestingly, ranking varied a lot, underlining that participants also prioritised other questions. It is possible that participants prioritised more realistic aims or short-term priorities that could be achieved in the near future. We unfortunately did not differentiate between short-term and long-term priorities in our survey.

Compared with healthcare professionals, patients with PCD and their caregivers identified many similar priorities, especially related to management of the disease and its symptoms.^23^ However, the ranking of priorities often differed; for example, mental health was ranked much higher by patients than professionals. Topics raised exclusively by patients were related to effects of medication and treatment burden, for example, antibiotic resistance, long-term impacts of PCD treatments and how treatment efforts can be reduced without compromising effectiveness. Several areas, which were identified as needing further research such as treatment burden, fertility, ENT disease and genotype-phenotype correlations, are topics of recent or planned studies. Some of these studies have extensive patient involvement showing that the PCD research community considers more and more the perspective of people with PCD and their families.^2^,^447^ Persons with PCD also ranked research on mental health and fertility higher than parents/caregivers of children with PCD. A systematic review and meta-analysis showed that living with a chronic disease can have an increasing impact on mental health even up to adulthood.^50^ A recent study on addressing fertility questions in PCD showed commonly delayed information on possible fertility issues, with many affected people with PCD becoming informed only when facing fertility struggles.^51^ It is possible that priorities of people living with a chronic disease shift with age and lived experiences are crucial in prioritising topics like mental health or fertility, while caregivers of children appear to be more focused on priorities related to treatments and disease management.

We found that people with PCD are motivated to participate in research when they are informed appropriately and invited. Their main research priorities relate to developing new treatments or improving the evidence base for existing treatments. Our findings contribute to improvement of future recruitment strategies and communication of research in an appropriate manner for the PCD patient community. Improved recruitment strategies and research communication will further encourage participation in future research. Jointly, priorities identified by people with PCD and PCD professionals, and continued collaboration with patient partners will undoubtedly help to plan more successful future research activities within the field of PCD.

## Supplementary material

10.1136/bmjresp-2025-003364 online supplemental file 1

## Data Availability

Data are available upon reasonable request.
