# Supplementary material for: Patients’ research priorities and participation in primary ciliary dyskinesia research
Source: BMJ Open Respir Res. 2025 Dec 14;12(1):e003364. doi: 10.1136/bmjresp-2025-003364 (PMC12706200; doi:10.1136/bmjresp-2025-003364)
Supplement: online supplemental file 1 [file bmjresp-12-1-s001.pdf]

Supplemental documents of "Patients' research priorities and participation in primary ciliary dyskinesia research"

Table S1: Participation in and knowledge of PCD patient support groups among survey participants (N=399)

Is there a patient support group in your country and are you involved in it?	N (%) Total	N (%) Countries with patient group[a]	N (%) Countries without patient group[b]
Yes, there is a patient group. I do not participate in it.	141 (35)	139 (38)	2 (5)
Yes, there is a patient group. I attend meetings and participate in activities.	107 (27)	101 (28)	6 (17)
Yes, there is a patient group. I am a representative/ committee member.	38 (10)	37 (11)	1 (3)
No, there is no patient group.	48 (12)	24 (6)	24 (67)
I don't know if there is a patient group.	65 (16)	62 (17)	3 (8)

PCD: primary ciliary dyskinesia. Characteristics presented as N (%). [a]Among 363 participants living in countries with known PCD support groups. [b]Among 36 participants living in countries which do not have PCD support groups.

Table S2:

Themes and specific questions related to research priorities for primary ciliary dyskinesia (PCD) from in-depth, semi-structured interviews with people with PCD or caregivers of children with PCD

Treatment strategies

1. Can we find a cure for PCD?
2. Can we test if CTFR modulator therapies work in PCD?
3. How can we improve ciliary beating everywhere in the body?
4. Can we test if Pulmozyme helps in PCD?
5. Can we test medications which are prescribed for PCD?
6. Can we test and compare inhalation devices for PCD?
7. Can we develop a physiotherapy app for PCD?
8. Can we develop a drug to reduce mucus production in PCD?
9. Can we develop a treatment for nasal polyps in PCD?
10. What are long-term treatment effects in PCD?
11. Can we develop individualised treatments by dosage and with different application routes for PCD?
12. Can we develop treatments to improve quality of life in PCD?
13. Can we stabilise lung function and reduce infections in PCD?
14. Can we develop new antibiotics?
15. Can we develop other new antimicrobic treatments to tackle antibiotic resistance?
16. When are intravenous or oral antibiotics more beneficial in PCD?
17. Can we test bronchiectasis treatments for PCD to reduce/reverse lung damage as tested in COVID-19 patients e.g. re-transfusion of selected white blood cells?

Symptoms

18. Can we study all symptoms related to PCD and look into differences by sex in PCD?
19. Can we study ENT symptoms and hearing problems in PCD?
20. Can we study gastrointestinal problems in PCD?
21. How is the genotype-phenotype correlation in PCD?
22. What are long-term disease effects in PCD?

Upper airways

23. Can we study effects of chronic rhinosinusitis on sinus surgery in PCD?
24. What is the benefit of sinus fenestration in PCD?
25. Can we develop hearing tests for home use to adjust hearing aids in PCD?
26. Can we study balance disorders in PCD?

Microbiology

27. What is the importance of certain pathogens in PCD?
28. What are long-term effects of taking antibiotics (e.g. on the gastrointestinal tract) in PCD?

Health-related behaviours/mental health

29. Can we study if sports or saline inhalation are better in mucus clearance in PCD?
30. Can we test if homeopathy or sauna reduce symptoms in PCD?
31. Can we study nutritional effects on symptoms in PCD in PCD?

32. Can we study how treatment duration affects daily life?
33. Can we research treatment burden in PCD?
34. Can we study psychological, psychosocial aspects and needed support in PCD?
35. How do people with PCD cope in daily life?
36. Can we study how nutrition and minimally needed fluid intake affect symptoms in PCD?

Comorbidities

37. Can we improve understanding of PCD to differentiate between other diseases?
38. Are learning difficulties associated with PCD?
39. Are dental abnormalities e.g. enamel issues associated with PCD?

Diagnosis

40. How can we improve diagnosis in PCD?

Research on special groups

41. How can we include more research including adults in PCD?

Management and care of PCD

42. Can we build PCD centre (for adults)?
43. How do we improve transition to adult care in PCD?
44. How can we increase PCD knowledge and management among medical staff to improve better treatment outcomes?
45. How can we improve multidisciplinary communication and treatment e.g. integration of treatment/management, social aspects in daily life in PCD?

Other

46. How is fertility affected and what are the best fertility management approaches in PCD?
47. Can we develop information (booklet) about PCD (what is PCD, how is it managed, what is the prognosis) – to have a clear guide to give to people or parents at diagnosis?
48. What causes fatigue in PCD and how can we treat it?
49. Can we study pain perception in PCD?

PCD: primary ciliary dyskinesia. CTFR: cystic fibrosis transmembrane conductance regulator. COVID: Coronavirus disease 2019

Table S3: Overall top research priorities for primary ciliary dyskinesia (PCD) as ranked by persons with PCD or parents/caregivers of children with PCD of survey participants.

Rank	Research topics	Total (n=374)	Persons with PCD (n=207)	Parents/Caregivers (n=167)
1	Can we find new medication that will 'cure' PCD or reduce the need for treatment by restoring the function of cilia in the body (like the medication available in cystic fibrosis)	0.511	0.430	0.550
2	Are there treatments that will improve lung function, reduce infections, and reduce the amount of mucus I produce?	0.241	0.170	0.202
3	What is the best way to treat PCD (including lungs, ears, and nose) using existing medication and other management approaches?	0.217	0.170	0.255
4	Can we find new antibiotics or other medication to tackle antibiotic resistance, and what are the effects of long-term antibiotic use?	0.137	0.140	0.093
5	How can we get more doctors and people with PCD involved in research and make them aware of this condition?	0.135	0.134	0.123
6	What health-related behaviours can I do, and what everyday things should I avoid to control the improvement or worsening of my symptoms and quality of life?	0.100	0.106	0.080
7	How is mental health affected in people with PCD and their families (psychological aspects of PCD e.g. treatment burden and coping in daily life)?	0.079	0.087	0.056
8	What is the life expectancy in PCD and what are the long-term impacts of this disease and its treatments?	0.060	0.067	0.039
9	How is fertility affected in patients with PCD and what are the best fertility management approaches?	0.059	0.068	0.033
10	What is the best medication plan for each patient with PCD, including specific dosages and route (orally or intravenous)?	0.058	0.041	0.055
11	How does PCD affect the ears, balance issues, nose, and sinuses and how are these problems linked with problems in the lungs?	0.052	0.052	0.044
12	How does PCD affect the gastrointestinal system, such as stomach and intestines (e.g. reflux, indigestion, bloating)?	0.050	0.062	0.035
13	Are specific genes associated with specific symptoms or more severe disease (genotype-phenotype correlation)?	0.047	0.038	0.056
14	How should PCD be managed correctly in different age groups (also in people without symptoms)?	0.044	0.033	0.070
15	How can the treatment duration and treatment efforts be reduced without compromising effectiveness?	0.042	0.039	0.040

Research topics ranked from most to least important (listed among top three overall priorities) based on the mean of reciprocal ranking score (0–1); each question was scored with 1 if ranked first, 1/2 if ranked second, 1/3 if ranked third, and 0 if not ranked among the top three priorities. PCD: primary ciliary dyskinesia. 25 participants preferred not reporting if they had PCD or were parents/caregivers of a child with PCD were excluded from the stratified score.

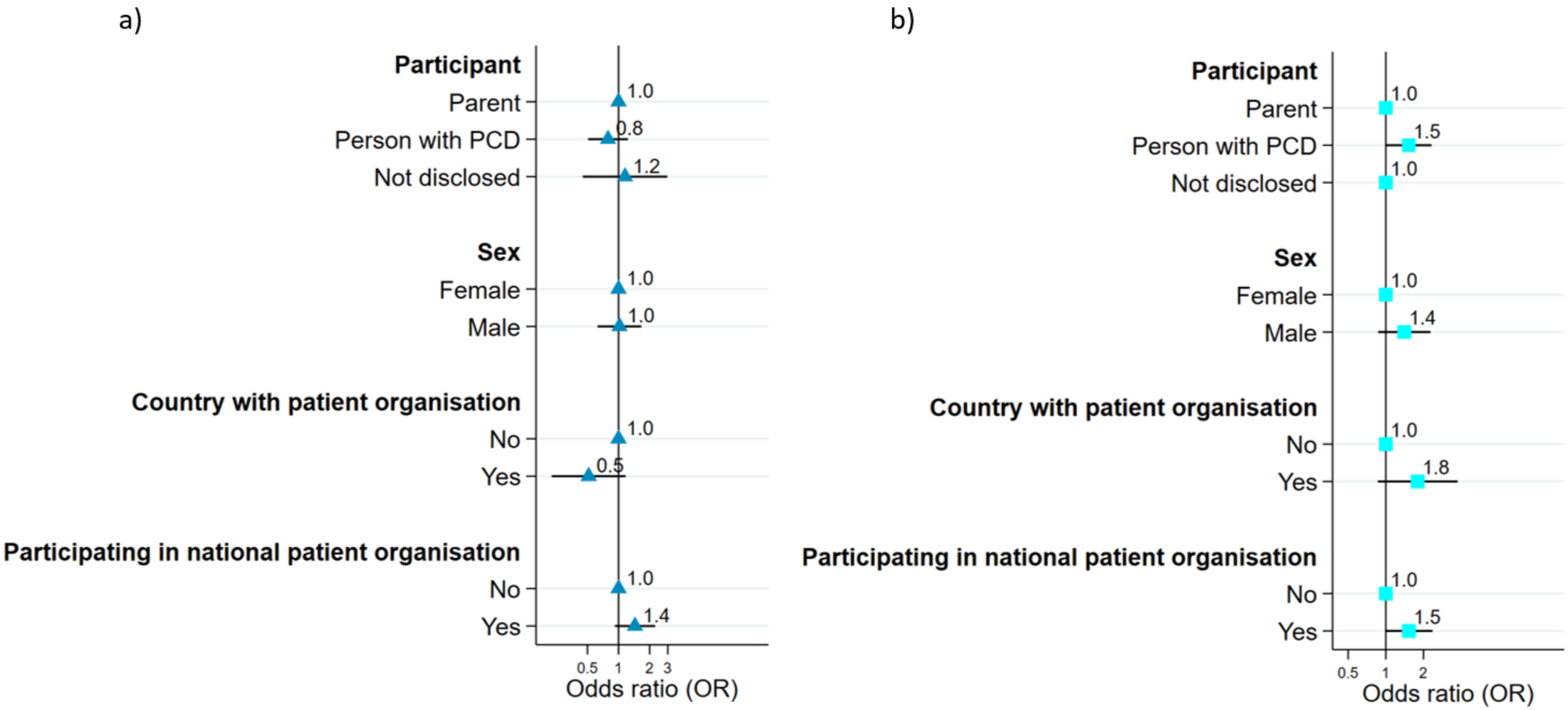

Figure S1: Participation of survey participants in research related to primary ciliary dyskinesia (PCD) N=399
a) Staying informed with PCD research b) Participation in PCD research
